# Targeting the Tumor Microenvironment for Improving Therapeutic Effectiveness in Cancer Immunotherapy: Focusing on Immune Checkpoint Inhibitors and Combination Therapies

**DOI:** 10.3390/cancers13061188

**Published:** 2021-03-10

**Authors:** I-Tsu Chyuan, Ching-Liang Chu, Ping-Ning Hsu

**Affiliations:** 1 Department of Internal Medicine, Cathay General Hospital, Taipei 10630, Taiwan; 144551@mail.fju.edu.tw; 2 Department of Medical Research, Cathay General Hospital, Taipei 10630, Taiwan; 3 School of Medicine, College of Medicine, Fu Jen Catholic University, New Taipei City 24205, Taiwan; 4 Graduate Institute of Immunology, College of Medicine, National Taiwan University, Taipei 100233, Taiwan; clchu01@ntu.edu.tw; 5 Department of Internal Medicine, National Taiwan University Hospital, Taipei 100225, Taiwan; 6 Department of Internal Medicine, College of Medicine, National Taiwan University, Taipei 100233, Taiwan

**Keywords:** cancer immunotherapy, immune checkpoint, T cell dysfunction, tumor microenvironment

## Abstract

**Simple Summary:**

Immune checkpoint inhibitors (ICIs) constitute a paradigm shift in cancer therapy, and it has greatly expanded our knowledge of anticancer immunity and has introduced breakthroughs in cancer therapy. However, despite the promising results in the use of immunotherapy in some cancers, numerous patients do not respond to ICIs without the existence of a clear predictive biomarker. This review provides an overview of recent advances in cellular and molecular factors within the tumor microenvironment (TME) to identify possible mechanisms of immunotherapy resistance, as well as to develop novel combination strategies for cancer immunotherapy. The in-depth exploration of complexity within the TME allows for the improvement of therapeutic efficacy and highlights its contribution to cancer immunotherapy.

**Abstract:**

Immune checkpoints play critical roles in the regulation of T-cell effector function, and the effectiveness of their inhibitors in cancer therapy has been established. Immune checkpoint inhibitors (ICIs) constitute a paradigm shift in cancer therapy in general and cancer immunotherapy in particular. Immunotherapy has been indicated to reinvigorate antitumor T-cell activity and dynamically modulate anticancer immune responses. However, despite the promising results in the use of immunotherapy in some cancers, numerous patients do not respond to ICIs without the existence of a clear predictive biomarker. Overall, immunotherapy involves a certain degree of uncertainty and complexity. Research on the exploration of cellular and molecular factors within the tumor microenvironment (TME) aims to identify possible mechanisms of immunotherapy resistance, as well as to develop novel combination strategies involving the specific targeting of the TME for cancer immunotherapy. The combination of this approach with other types of treatment, including immune checkpoint blockade therapy involving multiple agents, most of the responses and effects in cancer therapy could be significantly enhanced, but the appropriate combinations have yet to be established. Moreover, the in-depth exploration of complexity within the TME allows for the exploration of pathways of immune dysfunction. It may also aid in the identification of new therapeutic targets. This paper reviews recent advances in the improvement of therapeutic efficacy on the immune context of the TME and highlights its contribution to cancer immunotherapy.

## 1. Introduction

Accumulating results were reported by recent clinical trials involving cancer immunotherapy with various immune checkpoint inhibitors (ICIs)—including antibodies against cytotoxic T-lymphocyte-associated protein 4 (CTLA-4) and monoclonal antibodies (mAbs) against both programmed cell death-1 and the dysfunction/activation of cytotoxic T lymphocytes [1,2,3,4,5,6,7,8]. The blockade of immune checkpoints by ICIs reinvigorates dysfunctional T cells by restoring tumor-specific immunity for the eradication of cancer cells. Clear results for ICI therapy were documented for various cancers, including melanoma, lung cancer, renal cell carcinoma, and head and neck cancer. These findings clearly indicate the arrival of a new era of immunotherapy; the long-term control of cancer with durable treatment response now seems achievable. The use of mAbs greatly expanded our knowledge of anticancer immunity and introduced breakthroughs and new trends in cancer therapy.

The CTLA-4 is a counterreceptor for the B7 family such as CD80 and CD86 to outcompete with costimulatory molecules CD28 and transduces an inhibitory signal against T cell activation [9]. Ipilimumab, an mAb that targets CTLA-4, was approved as a checkpoint inhibitor for the treatment of advanced melanoma in 2011 [2]. Although the immune-related adverse events of anti-CTLA-4 mAb treatment are frequent and sometimes severe, anti-CLTA-4 treatment is the first proof-of-concept therapeutic strategy for checkpoint inhibition in cancer immunotherapy. PD-1 is another immune checkpoint that inhibits T cell activation by binding to its cognate receptor PD-1 ligand 1 (PD-L1) and PD-1 ligand 2 (PD-L2) [10]. Unlike CTLA-4, blockage of PD-1 or PD-1 ligand leads to less immune-related adverse events and therefore the subsequent development of immune checkpoint inhibitors mainly focus on targeting PD-1 or PD-1 ligand [6]. Since then, an mAb and immune checkpoint inhibitor that targets PD-1 or PD-L1 [11], was approved for use in immunotherapy. Furthermore, cell therapy using chimeric antigen receptor (CAR)-T cells was approved for clinical use [12,13]. These revolutionary changes in cancer immunotherapy led scholars to focus on cancer immunotherapy, especially in clinical trials, many of which are ongoing. Despite encouraging effects on some cancers, the clinical application of immunotherapy faces several challenges concerning its safety and efficacy. For example, according to one study, only a small subset of patients responds to PD-1 and CTLA-4 checkpoint inhibitors, and durable responses are observed in only approximately 20% to 40%, demonstrating the difficulty of predicting patient responses [14]. Moreover, individual variations in the immune response to tumors indicate the need to develop personalized immunotherapy that is based on biomarkers and to select more suitable therapeutic agents and combinations of strategies. Novel approaches for improving cancer immunotherapy focus on attaining results with greater safety and control, which not only extend the clinical indications of these therapeutic agents but also reduce the occurrence of immune-mediated adverse events. In particular, rational combination strategies could increase the efficacy of single-agent anticancer regimens, decrease individual dosage, enable more effective targeting, and attenuate the accumulation of off-target adverse effects. Ongoing research has centered on the development of novel combination strategies, delivery technologies, and specific targeting of the tumor microenvironment (TME) for cancer immunotherapy. These investigations have involved ICI-based combination therapies and have focused on first, improving anticancer drug delivery with nanoparticles and congregates and second, on developing treatments involving both neoantigen-specific CAR-T cells and combination treatments with metabolic inhibitors. The metabolic dysregulation within the TME hinders immunotherapy response but may provide key targets for more effective combinatorial approaches with ICIs. Given the dynamic nature and plasticity of the TME, the complexity of the TME must be analyzed to identify potential biomarkers, develop rational combinatorial approaches involving ICIs, and enhance the development of novel therapeutic agents with regard to cancer immunotherapy.

This paper provides a brief overview of the recent advances in treatments involving TME, with a focus on the use of ICI-based combination therapies to overcome the challenges concerning the clinical translation of immunotherapy. The current understanding of immunometabolism, the potential role of the gut microbiota as an ancillary indicator, and the status of combination cancer immunotherapy, are also presented.

## 2. Tumor Microenvironment and Cancer Immunotherapy with ICIs

The disruption of immune tolerance within the TME is the key mechanism of action of ICIs, and it determines the response in immunotherapy. The TME comprises various cell components, such as immune cells, endothelial cells and fibroblasts, and extracellular components that surround tumor cells fed by a complex vascular network. Within the TME, both innate immune cells (tumor-associated macrophages [TAMs], neutrophils, dendritic cells [DCs], myeloid-derived suppressor cells [MDSCs], and natural killer [NK] cells) and adaptive immune cells (T and B cells) interact with the tumor cells, either through direct contact or via chemokine and cytokine signaling pathways, thereby shaping the behavior and therapeutic response of the tumor. More importantly, immune cells could vary in their activation status and localization within the TME; moreover, they could exert synergic effects on or interfere with the efficacy of cancer immunotherapy.

Regarding TAMs, both M1 macrophages, which exert tumoricidal effects, and M2 macrophages, which promote tumorigenesis, are characterized by plasticity and reversibility, and the functional polarization of TAMs is primarily regulated by the TME [15]. TAMs also secrete cytokines, such as interleukin-10 and transforming growth factor-beta, that suppress T-cell proliferation and activation in the TME [16]. Myeloid-derived suppressor cells (MDSCs), a special heterogeneous population of myeloid-origin cells, comprise myeloid progenitor cells, immature macrophages, immature granulocytes, and immature DCs. MDSCs expand substantially during tumorigenesis, suppress T cell responses [17], and actively migrate to the tumor site, where they rapidly differentiate into TAMs. Tumor-associated endothelial cells (TECs) differ from normal endothelial cells in characteristics such as proliferation, migration, and responses to growth factors and chemotherapeutics. TECs not only modulate immune cell infiltration; TECs in the TME can specifically suppress T cell function through the expression of immune checkpoint ligands, such as PD-L1 and PD-L2 [18]. Studies demonstrated that the presence of CD8^+^ T cells is associated with the upregulation of immune inhibitory mechanisms that mediate immunosuppression [19]. Both preclinical and clinical trials established that the secretion of interferon (IFN)-γ by tumor-specific CD8^+^ T cells contribute to the upregulation of PD-L1 and, seemingly, PD-L2 on tumor cells [19,20,21] that ligate PD-1 on activated T cells, further inhibiting T-cell effector function [10]. One study observed a strong correlation of IFN-γ levels within the TME with PD-L1 expression in human tumors [19].

**Figure 1:**
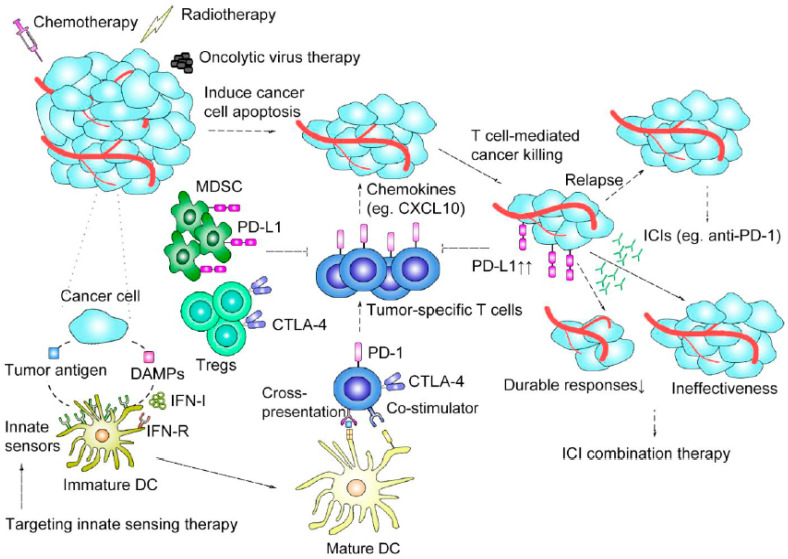
Immune-based mechanisms of immune checkpoint inhibitor (ICI) therapy and combination strategy rationales. Conventional cancer therapies, including chemotherapy, radiotherapy, and targeted therapy, induce cancer cell apoptosis as well as release tumor-associated antigens and various damage-associated molecular patterns (DAMPs) to activate dendritic cells (DCs) by triggering innate immune sensing pathways and type I interferons (IFN-I) ligating IFN receptors (IFN-Rs) on DCs. The interactions between cancer cells and activated DCs promote the cross-priming and recruitment (though chemoattractants such as CXCL10) of tumor-specific T cells for additional T cell–mediated killing of tumor cells. Conventional cancer therapies can also promote immunosuppression in the tumor microenvironment TME and cause resistance to immunotherapy by mechanisms involving the upregulation of PD-L1 on tumor cells and the accumulation of myeloid-derived suppressor cells (MDSCs) and regulatory T cells (Tregs). ICIs can restore the function of exhausted T cells or enhance that of cytotoxic CD8^+^ T cells to regain antitumor responses. Regarding ICI treatment failure attributable to initial ineffectiveness or the lack of durable responses, combination treatments involving the joint application of conventional therapies or immunotherapy can enhance the specific antitumor immune response, thereby achieving better tumor control. Oncolytic virus therapy, therapies targeting innate sensors, and other immunotherapies can strengthen the antitumor immune response.

Despite promising results in some specific indications, only a subset of patients respond to treatment for most cancers with PD-1 and CTLA-4 checkpoint inhibitors, and ICI therapy achieves durable responses only in approximately 20–40% of patients, exerting the greatest benefits in patients with melanoma and Hodgkin lymphoma [22]. A growing body of evidence indicates that some patients who initially respond to cancer treatment will ultimately experience drug-resistant relapse that leads to treatment failure and death in the span of months or years [23]. Mechanisms of resistance to ICI therapy include (1) a lack of tumor-infiltrating lymphocytes (TILs) and an increase in the following: recruitment of regulatory T cells (Tregs) into tumor sites, which makes less cancer killed or inadequate cytotoxicity, (2) immunosuppressive milieu within the TME, for which regulatory immune cells either by transducing negative signaling to inhibit cytotoxicity or secrete anti-inflammatory cytokines to build-up immune tolerance milieu, (3) tumor evasion of the immune system attributable to cancer cells’ lack of antigenicity, and (4) T cell dysfunction caused by the upregulation of alternative immune checkpoint ligands transducing negative signaling and lead to exhausted T cells (Figure 1).

Strategies for improving the success rate of ICI therapy focus on the identification of predictive biomarkers and new ICIs, the mitigation of toxicity effects, and the optimization of combination approaches that involve the simultaneous application of multiple checkpoints or using ICIs in concert with other agents. Predicting responsiveness to ICIs according to high-throughput data on the characteristics of tumor-infiltrating immune cells is considered a critical step in improving the success rate of ICI therapy and developing next-generation immunotherapeutic drugs for cancer.

Considerable research effort has been focused on applying moderate-resolution data pertaining to immune function in the TME that are sourced from low-resolution sources, such as immunohistochemical samples and microarrays of bulk tissue specimens. New analytical tools such as CIBERSORT [24] and xCell [25] use gene expression data from bulk tissue specimens to estimate the abundance of tumor-infiltrating immune cells. Immunoscore, an in vitro diagnostic test, uses a combination of immunohistochemical profiles and gene expression data in bulk tissue specimens to stratify patients by criteria concerning immune responses to cancer and thereby predict their prognosis [26]. All of these tools can estimate immunological frequency and cellular status in the TME; however, because of the disposition of the datasets used, they fail to reflect information related to actual cellular proportions and heterogeneity or deeper spatial distributions. Their contributions concern the classification of immune infiltrates in the TME, which is performed with the assistance of large databases and according to both the composition of these infiltrates and the characteristics of the inflammatory response. The enhancement of TME-targeted immunotherapy through the introduction of next-generation technologies will enable the exploration of deep sequencing in specific tumor types and subtypes and the prediction of the efficacy and response durability of existing ICIs; such enhancement may also yield innovations in cancer immunotherapy.

## 3. ICI Combination Therapy

**Figure 2:**
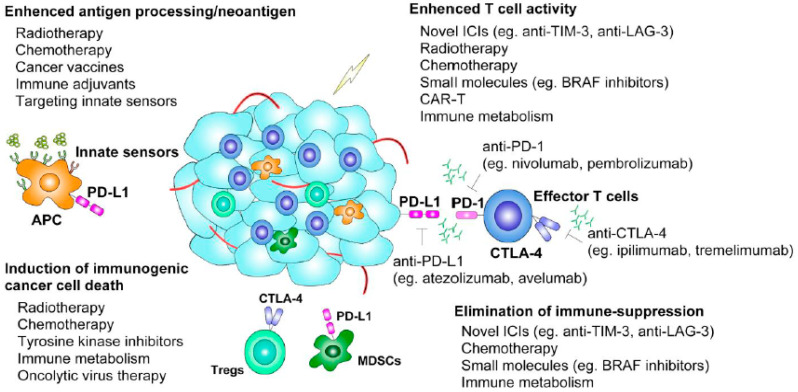
Summary of prospective combination strategies with checkpoint inhibition. Currently used monoclonal antibodies targeting PD-L1 (atezolizumab, avelumab), PD-1 (nivolumab, pembrolizumab), and CTLA-4 (ipilimumab, tremelimumab) as well as combination strategies involving various mechanisms for enhancing the efficacy of immune checkpoint inhibitors (ICIs) are presented. Novel immunotherapies, including oncolytic virus therapy and therapies targeting innate sensors, can either directly kill tumor cells or facilitate the presentation of tumor antigens in the generation of tumor-specific T cells. APC, antigen-presenting cell; BRAF, B-Raf proto-oncogene, serine/threonine-protein kinase; CAR-T cell, chimeric antigen receptor T cell; CTLA-4, cytotoxic T-lymphocyte-associated protein 4; LAG-3, lymphocyte-activation gene 3; MDSC, myeloid-derived suppressor cell; PD-1, programmed cell death protein 1; PD-L1, programmed cell death ligand 1; TIM-3: T cell immunoglobulin and mucin-domain-containing molecule 3; Tregs, regulatory T cells.

Many preclinical and clinical trials center on the approval of novel ICI combination regimens that aim to outperform monotherapy through the exploitation of synergistic effects. The strategies involve combining approved ICIs with other approved ICIs, newly identified checkpoints, cancer vaccines, TME-modulating agents, CAR-T cell therapy, immunotoxin therapy, or conventional radiotherapy and chemotherapy (Figure 2).

Anti-CTLA-4 mAb, an approved ICI, activates effector T cells by blocking the inhibitory signaling axis and depleting Tregs at the tumor site through antibody-dependent cellular phagocytosis or antibody-dependent cellular cytotoxicity. These processes are mediated by the binding of antibodies to the overexpressed CTLA-4 in the Tregs [27], which increases the ratio of CD8^+^ T cells to Tregs [28]. The main difference between CTLA-4 blockade and PD-1 blockade is in the targeted site (primarily in the TME and secondary lymph nodes, respectively) and the stage of inhibition (focused on the initial activation of naïve T cells and the specific targeting of activated T cells, respectively).

The effects of cancer immunotherapy are influenced by numerous factors. Regarding ICI therapy, both anti-PD-1/PD-L1 and anti-CTLA-4 mAbs appear to confer greater therapeutic benefits on patients with cancers with higher tumor mutational burden (TMB), which is regarded as an independent predictor of response to immunotherapy in various cancers, particularly melanoma and non-small-cell lung cancer [29,30]. However, a recent phase III clinical trial reported that TMB was not predictive of response to or benefits from the combination of atezolizumab (an anti-PD-L1 mAb) and chemotherapy [31]. Furthermore, CheckMate 214, another phase III clinical trial, reported no benefits in survival across PD-L1 levels under nivolumab–ipilimumab combination therapy for renal cell carcinoma [32]. The lack of appropriate biomarkers for predicting the therapeutic efficacy of ICIs and cancer immunotherapy and patient prognosis remains a critical need that must be addressed.

The characteristics of the TME constitute another factor that influences ICI efficacy. In the context of the TME, primary and metastatic tumors can be classified into “hot” and “cold” tumors. “Hot” tumors, which respond better to ICI therapy, are characterized by the high infiltration of T cells accompanied by the high expression of inhibitory checkpoints—such as PD-1, CTLA-4, and T cell immunoglobulin and mucin domain–containing molecule 3 (TIM-3)—and by the expression of lymphocyte-activation gene 3 (LAG-3). By contrast, “cold” tumors are considered immunologically “ignorant,” which means that they are characterized by neither antigenicity nor immunogenicity. This results from the rare expression of PD-L1, the high proliferation of cancer cells with low mutational burden, and the low expression of neoantigens [33]. The number of T cells that infiltrate such tumors is insufficient, meaning that they rarely respond to ICI therapy. This T-cell infiltrating gradient has been standardized as the immunoscore, which provides prognostic predictions that are more accurate than those made through the system of classification in terms of tumor size, node infiltration, and metastasis for malignant tumors. Anti-PD-1 mAb exerts no benefits on “cold tumors” because it is incapable of priming a sufficient number of the T cells already present within the tumor to launch antitumor responses. Although TILs are readily present in “hot” tumors with a high immunoscore, they are exhausted and express inhibitory-immune checkpoint molecules. Preclinical studies have noted the failure of anti-CTLA-4 mAb to restore the function of exhausted T cells. Moreover, if the T cells are not fully primed in advance, anti-PD-1 fails to induce full antitumor responses [22]. A phase III clinical trial reported that nivolumab–ipilimumab combination therapy was more effective for metastatic melanoma than single-target therapy was for PD-L1-negative melanoma [3]. In general, ICI combination therapy achieves durable responses in up to 60% of patients with metastatic melanoma; as for ICI monotherapy, durable responses were observed in 20–40% of patients [22].

Scholars are investigating various novel immune checkpoints. Studies have confirmed the presence of numerous checkpoint axes in exhausted TILs, which is referred to as the “compensatory upregulation of immune checkpoints.” In numerous types of tumors, blocking one immune checkpoint molecule leads to the upregulation of other such molecules, including CTLA-4, PD-1, TIM-3, and LAG-3 [34,35,36]. TIM-3 was originally described as an inhibitory receptor expressed on CD4^+^ and CD8^+^ T cells, and NK cells mainly bind to galectin-9 in antigen-presenting cells (APCs) to trigger T cell apoptosis [37]. TIM-3 can also bind to carcinoembryonic antigen-related cell adhesion molecule 1, which is expressed by various tumor cells and is upregulated by IFN-γ [38]. As a novel immune checkpoint that promotes peripheral tolerance, TIM-3 upregulates its expression in conjunction with chronic viral infections [39]. In one study, tumor-infiltrating, TIM-3-expressing CD4^+^ T cells exhibited an impaired capacity to produce IFN-γ and interleukin(IL)-2, but expressed higher levels of CD25 and forkhead box protein 3 (Foxp3) than levels of peripheral blood and non-TILs, suggesting that the TIL expression of TIM-3 contributes to the formation of the immunosuppressive TME [40]. An ex vivo study reported that over 80% of TILs isolated from tumors can coexpress PD-1, TIM-3, and LAG-3 [41]. Ongoing clinical trials are assessing the efficacy of combination therapy involving the blockade with anti-TIM-3 mAb and either anti-PD-1 or anti-PD-L1 in various advanced solid tumors. LAG-3, another novel checkpoint, is expressed on effector T cells, Tregs, and DCs, and the evidence indicates that the upregulation of both PD-1 and LAG-3 leads to T cell exhaustion and tolerance to tumor antigens [42]. The benefits of combination therapy with anti-LAG-3 and anti-PD-1 mAbs for various solid tumors is also being examined in clinical trials and awaits more mature clinical data.

Other novel immune checkpoints include T cell immunoreceptor with immunoglobulin and immunoreceptor tyrosine-based inhibitory motif domain (TIGIT), which is also expressed on activated CD4^+^ and CD8^+^ T cells, NK cells, follicular helper T cells, and natural killer T cells and is particularly highly upregulated in tumor-infiltrating Tregs [43]. Notably, in one study on advanced melanoma, TIGIT was upregulated and coexpressed with PD-1 in the majority of CD8^+^ TILs, and a dual blockade with TIGIT and PD-1 blockade additively increased proliferation, cytokine production, and degranulation in tumor antigen-specific CD8^+^ T cells [44]. A review article noted that preclinical studies established that anti-TIGIT and anti-PD-1 combination therapies enhance the expansion of CD8^+^ TILs and trigger cytotoxic responses to melanoma more effectively relative to checkpoint blockade alone [43]. Ongoing clinical trials are investigating monotherapy involving TIGIT blockade or combination therapy involving dual blockade with TIGIT and PD-1 in advanced solid cancers.

## 4. Cancer Immunotherapy and Anti-Angiogenic Drugs

The aberrant tumor vasculature is another critical factor that influences the immune responses in TME. For example, VEGF (vascular endothelial growth factor) secreted by tumors not only increases angiogenesis, but also modulates TCR signaling to inhibit T helper type 1 and cytotoxic T cell activity [19,33]. Several studies also demonstrated that VEGF inhibition could promote the differentiation and function of immune cells [19,45]. More and more evidence has shown that VEGF-A inhibition could increase the immune cell infiltration, providing a solid rationale for combining VEGF-targeted agents with immune checkpoint inhibitors. In renal cell carcinoma, several phase 3 clinical trials showed anti-VEGF or anti-VEGF receptor plus PD-1 or PD-L1 inhibition showed prolonged progression-free survival with favorable safety profile [46,47]. For highly immunosuppressed nature of microsatellite stable colorectal tumors, the use of anti-VEGF plus anti-PD-1 is inclusive; there is an international phase 2/3 clinical trial (CheckMate 9X8) is currently ongoing to evaluate effectiveness of nivolumab in combination with bevacizumab and FOLFOX (NCT03414983). Other cancers, including non-small cell lung cancer (adenocarcinoma) and hepatocellular carcinoma, also showed the positive results from several phase III studies of which combinations of PD-1/PD-L1 antibodies and anti-VEGF agents significantly improved clinical outcomes compared with respective standards of care [48,49,50], indicating targeting to angiogenesis is another key target for improving effectiveness of current cancer immunotherapy.

## 5. Combination of ICIs and Radiotherapy

Radiotherapy, either as monotherapy or as part of combination therapy, is used for the localized treatment of malignancies. According to a review article on cancers, the infiltration rate of activated memory CD4^+^ T cells and activated mast cells in the immune TME under radiotherapy are closely related to overall survival in multiple cancers [51]. In a mouse model of breast cancer, the combination of localized radiotherapy with CTLA-4 blockade for poorly immunogenic metastatic cancers refractory to anti-CTLA-4 monotherapy significantly improved survival [52]. This can be explained by the fact that anti-CTLA4 predominantly inhibits Tregs, thereby increasing the ratio of CD8^+^ T cells to Tregs, and that radiotherapy can further enhance the diversity of intratumoral T cells in the T-cell receptor repertoire [28]. Moreover, in a hypothetical phenomenon termed the abscopal effect, systemic antitumor reactions mediated by radiotherapy may lead to the regression of nonirradiated lesions. The combination of PD-1 blockade and radiotherapy can reinforce this effect [53], which is particularly observable in some hematologic malignancies [54]. In a phase III clinical trial on patients with locally advanced unresectable non-small-cell lung cancer, progression-free survival was significantly extended under consolidation therapy with durvalumab, an anti-PD-L1 mAb, after concurrent chemoradiotherapy relative to a placebo [55]. Retrospective safety analyses in patients with advanced solid tumors undergoing radiotherapy combined with anti-PD-1/PD-L1 blockade or radiotherapy and CTLA-4 blockade did not indicate increased toxicity [56]. Overall, the combination of radiotherapy with ICI therapy can sustainably strengthen anticancer immune responses with an acceptable safety profile. Furthermore, it contributes to a better prognosis.

## 6. Combination of Cancer Vaccines and ICIs

For decades, early studies on cancer vaccines had made little progress in cancer immunotherapy. Recent advances in the manipulation of immune cells through mRNA-based vaccines directly encoding neoantigens into patients are a novel strategy for cancer immunotherapy. Such neoantigen-encoding cassettes can include self-replication domains based on nonstructural protein machinery; the self-replicating double-stranded RNA in the cytoplasm can constitute a potent inducer for innate sensors, causing a strong immune response [57]. The advantage of the mRNA-based vaccine approach is that it can be highly personalized; specific neoantigens can be sequenced from individual patients and incorporated into the vaccine for delivery [58]. Such vaccines can be designed to deliver an abundance of antigens for simultaneous translation. They can also be part of the de novo design of personalized mRNA therapy for patients with cancer. The first application of mutant neoepitope-based vaccines in patients with melanoma was reported to significantly induce T cell infiltration and the cumulative rate of metastatic events; such an application also led to the neoepitope-specific killing of autologous tumor cells [58]. The shortage of cancer vaccines and the need for the rigorous development of various combinations of cancer vaccines and ICI therapy are attributable to the exhaustion of activated T cells. In a preclinical study of murine melanoma, the combination of a DNA-based vaccine with dual CTLA-4 and PD-1 blockade increased the intratumoral infiltration of CD8^+^ T cells [59]. Furthermore, in a mouse model of melanoma, vaccine-activated T effector cells potently synergized with a checkpoint blockade of CTLA-4 and PD-L1, leading to complete tumor regression, even under primary resistance to dual checkpoint blockade [60]. Clinical trials on applying mRNA cancer vaccines with anti-PD-1, anti-PD-L1, or anti-PD-L1 plus anti-CTLA-4 mAbs in solid tumors are ongoing.

## 7. Genetically Modified T-Cell-Based Adoptive Immunotherapy

T cells engineered to express a CAR focus on redirecting the effector activity of T lymphocytes toward tumor antigens. The adoptive cell transfer of CAR-T cells is a therapy approved for CD19^+^ B-cell malignancies, including B-cell acute lymphoblastic leukemia, non-Hodgkin lymphoma, and chronic lymphocytic leukemia. A review article indicated that multiple studies have investigated the effect of the use of CAR-T cells in solid tumors on suppressing the TME, regulating the homing of T cells to the tumor site, and enhancing the survival in and persistence of CAR-T cells in the tumor site [61]. The precise efficacy of CAR-T cell therapy in solid tumors remains unclear. However, the aim of adoptive cell transfer with chimeric receptors has been determined: the reduction of immune-related adverse events caused by the responses of CAR-T cells. Cytokine release syndrome, a common and well-understood toxicity of CAR-T cells, is characterized by the release of cytokines by infused CAR-T cells. Their infusion in turn promotes cytokine secretion in other immune cells, such as macrophages [62]. Notably, the occurrence of toxicity is accompanied by favorable T cell activity. Improvements to CAR-T cell therapy, which can be reasonably combined with other therapeutic approaches for the treatment of solid tumors, should be centered on the enhancement of safety and efficacy. Checkpoint blockade may strengthen the effects of CAR-T therapy in the hostile TME, as indicated in studies that have applied PD-1 blockade and CAR-T cell therapy in concert [63,64]. Another combination strategy involves the use of engineered CAR-T cells with self-secreting anti-PD-1 and the silencing of PD-1 expression to redefine their combination. A study reported that PD-1 blockade with the continuous secretion of anti-PD-1 blocked inhibitory checkpoint signaling and restored expansion and effector functions in T cells both in vitro and in vivo [65]. The efficacy was comparable or even superior to that achieved under the direct combination of CAR-T cell therapy and PD-1 blockade, because the secreted PD-1 can be better retained in the tumor site and protect CAR-T cells from PD-1 activation [66]. In a recent study, PD-1 disruption in epidermal growth factor receptor variant III–targeting CAR-T cells was accomplished through a method involving clustered regularly interspaced short palindromic repeats (CRISPR)/CRISPR-associated protein 9 [67]. Drawing from findings from preclinical studies, several ongoing clinical trials are using novel technology with regard to combination therapy with CAR-T cells and PD-1 blockade, mostly to treat B-cell lymphomas.

## 8. Immune Metabolism in Cancer Immunotherapy

At this point, it is clear that metabolic reprogramming is a hallmark of cancer progression. Specifically, cancer cells undergo major changes in metabolism to meet biosynthetic and bioenergetic requirements for rapid proliferation under and adaptation to the stressful conditions of the TME. A key mechanism of cancer treatment resistance involves the metabolic reprogramming and plasticity of cancer cells [68]. However, it is not only cancer cells that undergo metabolic alterations to promote cell proliferation; the metabolic reprogramming of immune cells, such as TAMs, MDSCs, and T cells, governs the balance of the immune TME and the effectiveness of cancer immunotherapy. Through the maintenance of active glycolytic metabolism, TAMs contribute to tumorigenesis-inducing cancer-mediated inflammation by producing reactive nitrogen species, reactive oxygen species, and inflammatory cytokines, such as tumor necrosis factor and interleukins 1 and 6 [69]. MDSCs are functionally characterized by their potent immunosuppressive effects on tumor-infiltrating T cells, the mechanisms of which mainly involve the depletion of amino acids and the production of oxidative stress mediators, such as reactive nitrogen species and reactive oxygen species [70]. The crosstalk of T cells with innate immune cells and cancer cells regulates the metabolic reprogramming of tumor-infiltrating T cells in the TME, where the lack of nutrients contributes to the high metabolic activity of cancer cells and the poor vascular blood flow. Impairments to the signaling of the T-cell receptor and to glycolytic and amino acid metabolism in turn result in impaired antitumor effector function in tumor-specific T cells [71]. Furthermore, hypoxia in the TME can induce the expression of hypoxia-inducible factor 1-alpha and promote both Treg generation and maintenance and PD-L1 expression in MDSCs, thereby exerting potent immunosuppressive effects on tumor-specific effector T cells [72].

An increasing number of studies have linked ICI resistance with immune metabolism. In one study involving two immunotherapeutic mouse models, obesity-induced leptin limited ICI response, whereas leptin receptor blockade improved ICI response [73]. In another study, a better response rate and a longer time-to-treatment-failure were observed in patients with various cancers and with overweight or obesity undergoing ICI therapy. Progression-free survival and overall survival were also enhanced [74]. In a mouse tumor model, mitochondrial activation chemicals such as reactive oxygen species precursors or mitochondrial uncouplers synergized with PD-1 blocking immunotherapy, improving antitumor responses [75]. In a study involving mouse melanoma models, the combination of PD-1 blockade with the pharmacologic induction of fatty acid catabolism by fenofibrate preserved the effector function of CD8^+^ TILs and slowed tumor progression [76]. Although the research remains nascent, the combination of cancer immunotherapy with immunosuppression-mediating metabolic targets should be explored in the development of approaches that increase immunotherapy efficacy.

## 9. Cancer Immunotherapy and the Gut Microbiota

A growing body of evidence has indicated that gut microbiota is closely related to the pharmacological efficacy of cancer immunotherapy. The gut microbiota, which shapes therapeutic effectiveness through metabolic manipulation, enzymatic degradation, and translocation, in particular the reduction of its diversity and ecological variability, may constitute a suitable target for promoting the efficacy and reducing the adverse effects of cancer immunotherapy. In a mouse model of melanoma, commensal *Bifidobacterium* contributed to the differences in responses to ICIs, and the antitumor efficacy of PD-L1 was enhanced through fecal microbiota transplantation (FMT) [77]. A study on patients with advanced cancers reported that first, antibiotics reduced ICIs’ clinical benefits and second, FMT from cancer patients who responded to ICIs into germ-free or antibiotic-treated mice ameliorated the antitumor effects of PD-1 blockade [78]. Furthermore, a metagenomic analysis of the stool of patients with cancer at diagnosis revealed positive correlations between clinical responses to ICIs and the abundance of *Akkermansia muciniphila*. Additional oral supplementation with *A. muciniphila* after FMT with nonresponder feces effectively restored PD-1 blockade efficacy [78]. A study involving 16S rRNA gene sequencing of oral and fecal samples from patients with melanoma treated with anti-PD1 showed that *Faecalibacterium* was positively correlated with progression-free survival, whereas *Bacteroidales* increased the risk of relapse [79]. Similarly, a study noted that the antitumor effects of CTLA-4 blockade were dependent on specific *Bacteroides* species [80]. Overall, the diversity of microbiota and the presence of specific bacterial species or genera (at least *A. muciniphila*, *Bifidobacterium*, and *Faecalibacterium*) contribute to the antitumor immune responses of ICIs. Given the clinical correlation of the microbiome with ICI efficacy, microbiome profiling can not only serve as a marker for successful response, but also support the use of probiotics as an adjunct to ICI therapy. A study posited that from a mechanistic standpoint, ICI efficacy may be ascribable to the bacteria in the TME. For example, *Fusobacterium nucleatum* in the TME can directly bind with and inhibit the activity of natural killer T cells and T cells via the immunosuppressive TIGIT receptor [81]. Overall, in the literature, clinical and preclinical evidence suggests that gut microbial abundance plays a regulatory role in tumor therapy, particularly through the enhancement of sensitivity to immunotherapy. Microbiome-targeted probiotic interventions may have potential for application in patients with cancer who are resistant to ICI therapy.

## 10. Conclusions

The development of cancer immunotherapy represents a paradigm shift in the treatment of cancer. Immunotherapy has been indicated to reinvigorate antitumor T cells and dynamically modulate anticancer immune responses. Encouraging improvements in the efficacy of immunotherapy and the reduced occurrence of adverse events were noted in the pursuit of new targets and methods for cancer treatment (e.g., combination therapy). Nevertheless, immunotherapy remains complex and uncertain. Ongoing research is exploring cellular and molecular factors within the TME to identify possible mechanisms of immunotherapy resistance and to develop TME-targeted treatment as well as novel combination strategies in the context of cancer immunotherapy. The combination of cancer immunotherapy with other types of treatment, including immune checkpoint blockade therapy involving multiple agents, most responses and effects in cancer therapy could be significantly enhanced, but the appropriate combinations have yet to be established. These approaches require the identification of better predictive biomarkers to determine the immune status of specific tumors through the refinement of liquid biopsies and through immune positron emission tomography, tumor bulk deconvolution, and single cell approaches [33]. Moreover, new combination strategies, whether they involve cancer vaccines and CAR-T or supplementation with agents targeting the metabolism and the gut microbiota, exhibited potential in both preclinical models and early-stage clinical trials. A clearer understanding of the critical and fundamental immune mechanisms of unresponsiveness and resistance to existing cancer immunotherapy will provide greater insight into treatment design and development.

## Data Availability

Data available in a publicly accessible repository.

## Abbreviations

APC: antigen-presenting cell; CAR-T cell, chimeric antigen receptor T cell; CTLA-4, cytotoxic T-lymphocyte-associated protein 4; DC, dendritic cell; IFN, interferon; ICI, immune checkpoint inhibitor; LAG-3, lymphocyte-activation gene 3; MDSC, myeloid-derived suppressor cell; NK cell, natural killer cell; PD-1, programmed cell death protein 1; TME, tumor microenvironment; TIGIT, T cell immunoglobulin with immunoglobulin and immunoreceptor tyrosine-based inhibitory motif domain; TIL, tumor-infiltrating lymphocyte; TIM-3, T cell immunoglobulin and mucin-domain-containing molecule 3; Treg, regulatory T cell.
